# Untangling sporadic brain arteriovenous malformations: towards targeting the KRAS/MAPK pathway

**DOI:** 10.3389/fsurg.2024.1504612

**Published:** 2024-12-02

**Authors:** Rashad Jabarkheel, Lun Li, Maxwell Frankfurter, Daniel Y. Zhang, Avi Gajjar, Najib Muhammad, Visish M. Srinivasan, Jan-Karl Burkhardt, Mark Kahn

**Affiliations:** ^1^ Department of Neurosurgery, Perelman School of Medicine, University of Pennsylvania, Philadelphia, PA, United States; ^2^ Department of Medicine and Cardiovascular Institute, University of Pennsylvania, Philadelphia, PA, United States

**Keywords:** arteriovenous malformation, sporadic, KRAS, MAPK, MEK inhibitor

## Abstract

Brain arteriovenous malformations (AVMs) are vascular lesions characterized by abnormal connections between parenchymal arteries and veins, bypassing a capillary bed, and forming a nidus. Brain AVMs are consequential as they are prone to rupture and associated with significant morbidity. They can broadly be subdivided into hereditary vs. sporadic lesions with sporadic brain AVMs representing the majority of all brain AVMs. However, little had been known about the pathogenesis of sporadic brain AVMs until the landmark discovery in 2018 that the majority of sporadic brain AVMs carry somatic activating mutations of the oncogene, *Kirsten rat sarcoma viral oncogene homologue* (*KRAS*), in their endothelial cells. Here, we review the history of brain AVMs, their treatments, and recent advances in uncovering the pathogenesis of sporadic brain AVMs. We specifically focus on the latest studies suggesting that pharmacologically targeting the KRAS/MEK pathway may be a potentially efficacious treatment for sporadic brain AVMs.

## Introduction

**Figure 1:**
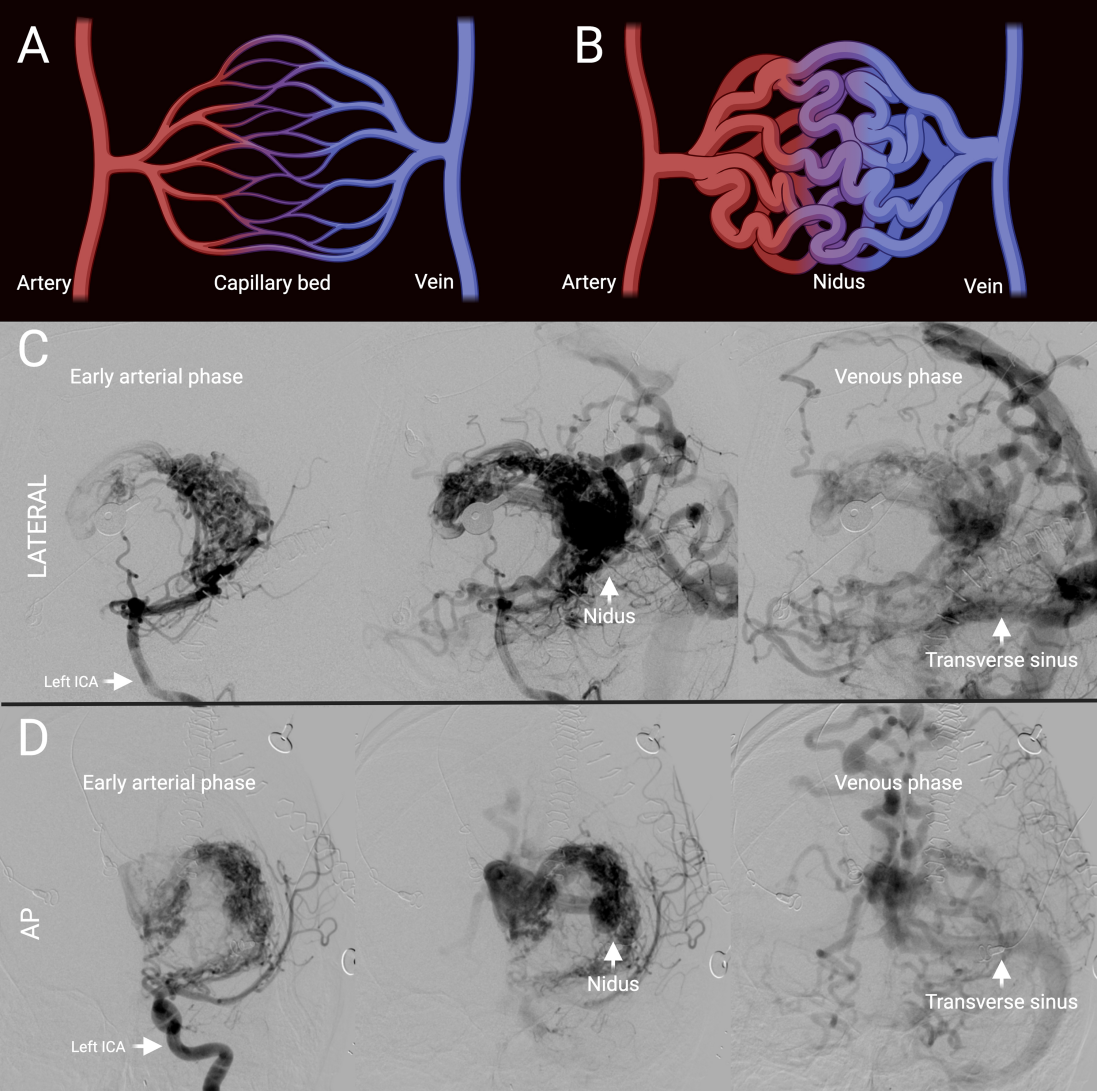
Representative brain AVM. **(A)** Schematic of normal arteriovenous connection via a capillary bed. **(B)** Schematic of AVM with absence of intervening capillary bed and instead presence of a nidus. **(C)** Lateral projection of cerebral angiogram of representative Spetzler-Martin grade 5 AVM showing early arterial to venous phase from left to right. **(D)** Anterior-posterior projection of cerebral angiogram of representative Spetzler-Martin grade 5 AVM previously shown in **(C)** showing early arterial to venous phase from left to right. ICA, internal carotid artery; AP, antero-posterior.

Brain arteriovenous malformations (AVMs) are vascular lesions characterized by an abnormal, fistulous connection between parenchymal arteries and veins without an intervening capillary network (1) (Figure 1). AVMs are prone to rupture due to the direct shunting of high pressure arterial blood into veins designed to carry low pressure post-capillary blood with an annual risk of rupture of 2%–4% and a lifetime risk of rupture of 105 minus the patient's current age in years (2, 3). In addition to intracranial hemorrhage (ICH), AVMs can present with seizures, headaches, or a variety of other neurologic deficits related to ischemic steal from adjacent brain parenchyma (2). The best estimate of the incidence of AVMs is 0.94 per 100,000 person-years with the most common presentation being ICH in nearly half of all patients (4–10). The consequences of hemorrhage are devastating. Approximately 8% of patients die in hospital after a single AVM hemorrhage event. Among survivors, 46% are discharged from the hospital with mild to moderate neurologic deficits, while 23% have severe neurologic deficits (11). The neurologic deficits sustained as a result of AVM hemorrhage are associated with significant disability-adjusted life years as AVMs typically present in young adults between the ages of 20–40 (12).

Treatment of AVMs is challenging and largely dependent on their Spetzler-Martin grade. The Spetzler-Martin grading scale scores AVMs based on the size of their nidus, functional location within the brain, and pattern of venous drainage (13). The Spetzler-Martin grade is clinically consequential as it predicts the rate of favorable outcome following any interventional treatment for a given AVM. Grade 1 and 2 AVMs are generally treated with curative microsurgery and have favorable outcomes (14). Of note, the more recently developed Lawton-Young supplement to the Spetzler-Martin grading scale helps to further stratify surgical risk for operable AVMs by also taking patient age, nidus architecture, and whether a patient presented with hemorrhage into account (15). Management of grade 3 AVMs is more nuanced and typically involves a multi-modal approach involving radiosurgery, endovascular embolization, and microsurgery (16). Grade 4 and 5 AVMs are generally managed conservatively as any treatment is associated with high rates of morbidity and mortality (17). Some high grade AVMs can be downgraded with radiosurgery or endovascular embolization to make definitive surgery an option (18). It is difficult to estimate the exact prevalence of Grade 4 and 5 AVMs as most are not intervened upon, however, based on data from historical surgical series, in which Grade 4 and 5 AVMs represented 20% of all surgically treated AVMs, it is likely that they comprise at least a third of all AVMs (17). Importantly, no pharmacologic treatments are currently approved to reduce the size or risk of hemorrhage of brain AVMs.

Given the disqualifying morbidity associated with contemporary treatment options for high grade AVMs, and the intrinsically invasive nature of surgery for low grade AVMs, there is a critical need for the development of targeted pharmacologic therapies for these complex lesions. Over the last decade significant progress has been made in delineating the genetic basis of AVMs such that targeted therapeutics for these complex lesions are now in sight.

## A brief history of brain AVM treatment

To envision the future of brain AVM treatment, we must first understand the history of treatment for these complex lesions. Brain AVMs were first reported in the mid 19th and early 20th century with credit primarily given to either Hubert von Luschka or Rudolf Virchow for the seminal report in the literature (19, 20). Early descriptions of brain AVMs were challenged by lack of photography and much less advanced imaging such as computed tomography (CT), magnetic resonance imaging (MRI), or catheter based angiography, making it difficult to distinguish true nidal AVMs from similar vascular pathology such as dural arteriovenous fistulas, slow flow vascular malformations such as cavernomas, and hypervascular tumors such as hemangioblastomas (21). Harvey Cushing and Percival Bailey in their landmark 1928 manuscript, *Tumors Arising from the Blood-vessels of the Brain*, demarcated AVMs, also known as “angioma arteriale” at the time, as unique within the subclassification of “angiomatous malformations” and separate from “angioblastomas”, or hypervascular tumors (21).

Early attempts at surgical resection of AVMs by the great American pioneers of neurosurgery, Harvey Cushing and Walter Dandy, who separately published their individual cases series in 1928, were met with abysmal results (21, 22). Cushing had the following to say about the surgical management of AVMs at the time (21):

“There is little to be said from our own experience in any way encouraging in regard to the surgical attack on one of these formidable lesions even in the absence of any participation of the extracranial vessels in the increased vascularity. Elsewhere in the body where tourniquets could be applied or pressure be temporarily exerted against resistant tissues, bleeding from an angioma can be controlled, but this is not true of the brain. Forewarned is to be forearmed; and since an angioma is likely to be exposed during the course of an exploratory operation for a lesion of unknown character, the surgeon is quite unprepared to meet the situation. Indeed, as the cases reported in the literature bear evidence, the very attempt to uncover the lesion may be disastrous.”

The first successful removal of an AVM was ultimately performed in 1932 by the father of Swedish neurosurgery, Herbert Olivecrona (23). Olivecrona's technique for AVM removal, consisting of circumferential ligation of feeding arteries in a cone shaped fashion towards the depth of an AVM before ligating any draining veins, is the basis for present-day AVM resection technique (23, 24). Olivecrona's improved outcomes with surgical resection of AVMs was due not only to technique, but also due in part to the rise of cerebral angiography, which was developed in 1927 by the Portuguese neurologist, Egas Moniz, and allowed for improved pre-operative characterization of AVMs (25, 26). The next major advancement in the surgical management of AVMs came with the introduction of the microscope to neurosurgery in the 1960 s and −70 s (24, 27). While in subsequent decades there have been further refinements in the pre-operative evaluation of AVMs with the advent of advanced CT and MRI imaging techniques, in addition to further improvements in cerebral angiography, the fundamental tenets and limits of surgical resection of AVMs have not changed dramatically since the 1980 s (24, 26). Presently, microsurgery is an effective albeit invasive option for treating low grade AVMs with minimal morbidity, however, it carries significant morbidity for treating high grade AVMs (15, 17, 18).

The first use of radiation to treat a brain AVM was in 1914 by Norwegian neurosurgeon Vilhelm Magnus (28). Conventional fractionated radiotherapy, which was the primary form of radiation therapy for treating AVMs for most of the 20th century, was largely ineffective (24, 29, 30). With the development of stereotactic radiosurgery in the 1970s by Swedish neurosurgeon and Olivecrona disciple, Lars Leksell, there was renewed interest in treating AVMs with radiation (30). By the early 1990s, reports out of the University of Pittsburgh, the site of the first stereotactic gamma knife unit in the United States, showed high rates of obliteration with gamma knife treatment of small volume AVMs (30–32). In subsequent decades despite attempts to expand the indications for radiosurgery in the management of AVMs its role has remained relatively limited at most centers (33). One of the major drawbacks of radiosurgery is that its mechanism of action, intimal hyperplasia, occurs in a delayed fashion and it may take between 1 and 3 years to achieve AVM obliteration, during which time the risk of hemorrhage persists (33, 34). Another major drawback of radiosurgery is that the larger the AVM is, the lower the marginal dose of radiation that can be safely delivered without damaging adjacent brain parenchyma (33). Presently, stereotactic radiosurgery serves as (1) a modestly less efficacious but also less invasive alternative to surgery in patients with low grade AVMs located in either deep or eloquent locations, (2) a provisional option for downgrading high grade AVMs for possible surgery, and (3) as a palliative option for high grade AVMs (18, 33).

The first attempted endovascular embolization of a brain AVM was performed in 1959 by American neurosurgeons Alfred Luessenhop and William Spence (35). Luessenhop and Spence cannulized the internal carotid artery at the carotid bifurcation, and then released methyl methacrylate emboli, measured to be just smaller than the approximate diameter of the patient's major AVM feeding artery. Importantly, their technique for AVM embolization relied on the imperfect hypothesis that the increased size and flow through an AVM's feeding arteries would selectively attract appropriately sized emboli that were released from the cervical internal carotid artery (35). Endovascular neurosurgery has come a long way since the time of Luessenhop and Spence. Numerous advancements have been made with the development of sophisticated catheters allowing for superselective targeting of vessels, improved embolization materials allowing for robust filling of involved vessels, and innovative transvenous embolization techniques (24, 36, 37). Despite these advances, however, angiographic AVM obliteration remains difficult to achieve and is associated with high morbidity, thus the role of endovascular approaches in AVM management remains limited (24, 38–41). Presently, the role of endovascular embolization at most centers is confined to either being (1) a pre-operative surgical adjunct by targeting deep feeding arteries to make surgery more facile, or (2) a salvage option in unresectable lesions by targeting high-risk features, such as intra-nidal aneurysms (38, 40).

## The genetics of brain AVMs

Given the limitations of surgical, radiosurgical, and endovascular treatments for AVMs, as described above, a better understanding of the underlying pathophysiology of AVMs is needed to provide a potentially pharmacological treatment option. Until recently however the pathogenesis of AVMs has been poorly understood. Since their first descriptions in the literature the prevailing paradigm has been that AVMs are static, non-neoplastic, congenital anomalies (12, 18, 42–45). In recent decades, as components of this paradigm have come into question, and DNA sequencing techniques have advanced, a more dynamic view of AVMs, as active lesions, secondary to aberrantly functioning vascular endothelial cells has emerged, presenting opportunities for pharmacologic intervention (42, 46, 47).

It has been dogma in neurosurgery that AVMs are congenital anomalies (44, 48). The consensus has been that AVMs develop at some point during embryogenesis, due to some unknown error in brain vasculogenesis, such that they are present at the time of birth, remain dormant until young adulthood, and only become apparent either once an AVM becomes symptomatic, or is incidentally found during cranial imaging for another indication (12, 45). The notion that AVMs are congenital stemmed from the early observation that AVMs present in young adults. Cushing noted in his manuscript, *Tumors Arising from the Blood-vessels of the Brain*, “the fact that the first symptoms have usually occurred in young adult life may be taken as in favour of a congenital lesion which for a period of years has remained symptomatically dormant” (21). The concept of AVMs as congenital anomalies carried with it the underlying assumption that these lesions are static, irreversible, developmentally primitive shunts, rather than active, proliferative centers of vascular dysfunction. Despite a long held consensus that AVMs are congenital there has been limited evidence to support this theory (42, 44). For instance, despite advances in the quality of fetal imaging techniques, and the frequency with which they are obtained, prenatal diagnosis of parenchymal AVMs is extremely rare (44, 49, 50). Moreover, as MRI studies are obtained with increasing frequency in contemporary medicine, increasing numbers of *de novo* cases of brain AVMs are being reported (42, 44, 51, 52). Taken together, these findings have opened the possibility that AVMs may be more than just one-off errors in embryonic brain vasculogenesis (53).

The first major breakthrough in uncovering the pathogenesis of brain AVMs came in the 1990s with the discovery of the genetic basis of Rendu-Osler-Weber disease, also known as hereditary hemorrhagic telangiectasia (HHT) (54–56). Rendu-Osler-Weber disease was first described in the late 19th century, but the term HHT wasn’t coined until 1909 (57). HHT was initially described as a familial disease characterized by epistaxis, mucocutaneous telangiectasias and anemia (57, 58). For many decades an association between HHT and brain AVMs was not well appreciated, as the overwhelming majority of brain AVMs are not familial, and only a small fraction of HHT patients develop brain AVMs (56, 58–61). Specifically, familial brain AVMs are estimated to represent approximately 5% of all brain AVMs, with the rest being sporadic, and only about 10% of HHT patients ultimately develop brain AVMs (48, 56, 62, 63). The finding that HHT is caused by germline, loss of function mutations in either *Endoglin* (*ENG*), *Activin receptor-like kinase 1* (*ALK1*), or *Mothers against decapentaplegic homolog 4 (SMAD4)* was of particular significance because all three are involved in endothelial cell specific receptor pathways of the transforming growth factor beta (TGF-β) family of ligands, suggesting that vascular endothelial cell dysfunction might be the driving force behind AVM development (56, 64, 65). In the following decades since the turn of the millennium, numerous transgenic HHT mouse models have been developed to study AVM pathogenesis and collectively have shown that (1) vascular endothelial cells are indeed the principal drivers within the neurovascular unit of AVM development, (2) ENG, ALK1, and SMAD4 promote quiescence within vascular endothelial cells, (3) AVMs can be induced by genetic manipulation at either the early postnatal or adult timepoint, and (4) mosaic genetic perturbation of endothelial cells is sufficient to cause AVMs (64–67).

**Figure 2:**
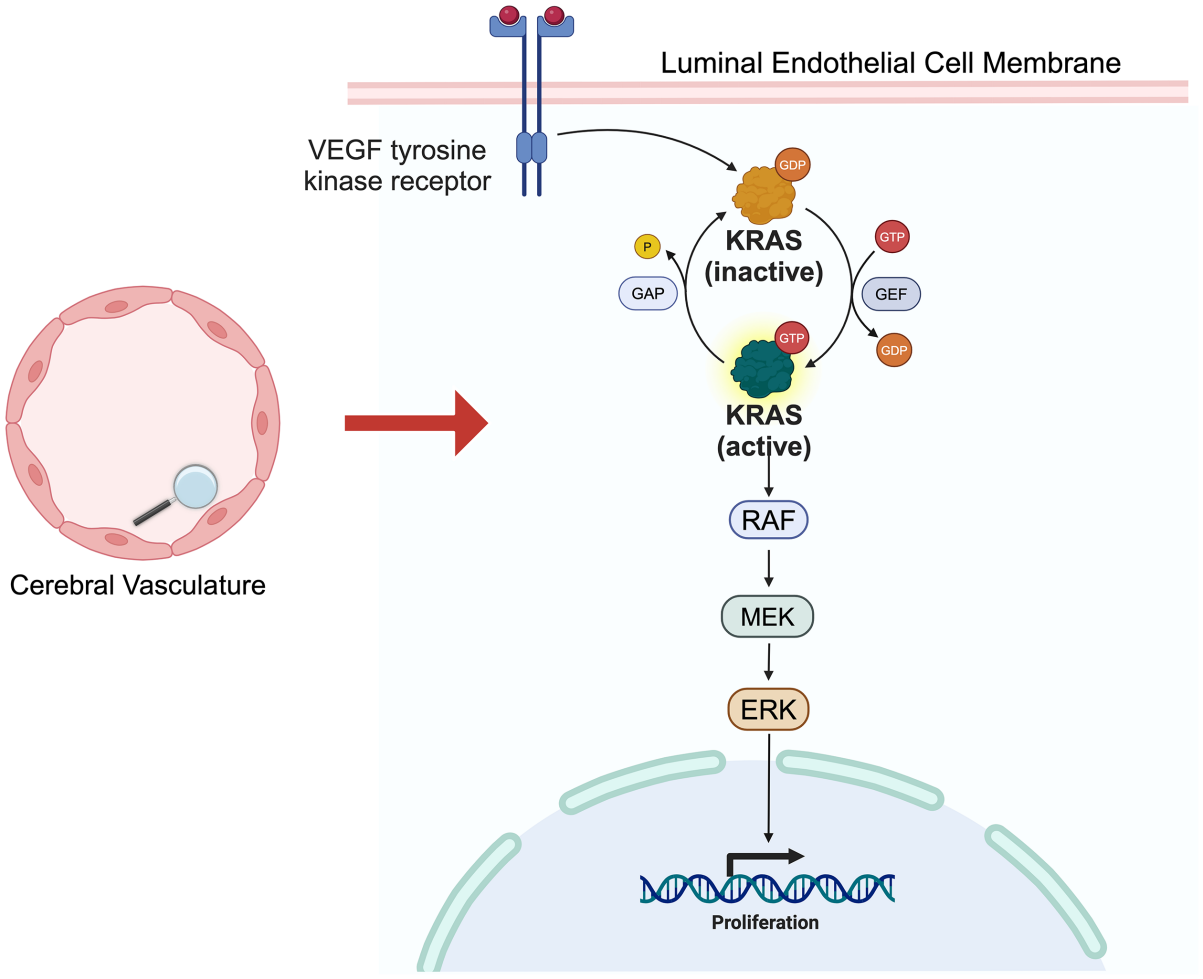
KRAS/MEK pathway. Simplified schematic of KRAS/MEK pathway. VEGF receptor activation triggers KRAS activation, followed by RAF, MEK and ERK activation, ultimately leading vascular endothelial cell proliferative changes.

Uncovering of the genetic basis of HHT spurred novel insights into the pathogenesis of hereditary AVMs, however, the generalizability of these findings to the pathogenesis of sporadic AVMs remained indeterminate. Beyond the fact that hereditary AVMs represent only 5% of brain AVMs, they are also more likely to be low grade and may have a lower risk of rupture compared to their sporadic counterparts (63, 68–70). Nonetheless, the discovery that hereditary AVMs were driven by germline mutations affecting brain vascular endothelial cell function led to the novel hypothesis that sporadic AVMs may in a parallel fashion be due to acquired somatic mutations of brain vascular endothelial cells (63). In 2018, a landmark study, which involved paired exome sequencing of human brain AVM and blood samples, found that most human brain AVMs carry somatic activating mutations of the oncogene, *Kirsten rat sarcoma viral oncogene homologue* (*KRAS),* in their endothelial cells (47). A high prevalence of *KRAS* mutations was subsequently validated in multiple other large cohorts of human brain AVMs (71–73). Building upon this work, it has recently been shown that either early postnatal or adult induction of activating *KRAS* mutations in brain endothelial cells is sufficient to induce formation of AVMs in transgenic mouse models (74–76). Much work remains to further elucidate the specific mechanisms by which activating *KRAS* mutations and consequent downstream stimulation of the mitogen-active protein kinase (MEK) and extracellular signal-related kinase (ERK) pathway leads to the development brain AVMs, however, it is known that this pathway is broadly involved in cell proliferation, as evidence by its involvement in up to 50% of all human cancers (77) (Figure 2). Another question which requires further investigation is how exactly loss of function mutations in the ENG/ALK1/SMAD4 pathway converge with gain of function mutations in the KRAS/MEK pathway to similarly confer an AVM phenotype (78).

## Future of brain AVM treatment

Since the establishment of activating *KRAS* mutations as the genetic basis of sporadic human brain AVMs there has been a push to apply therapeutics, initially designed to target dysregulated KRAS pathway signaling in the context of cancer, to AVMs (79). The hope is that by targeting aberrant KRAS pathway signaling in AVMs we may be able to either (1) induce complete regression of these lesions, (2) induce partial regression to make surgery feasible, or (3) reduce the risk of hemorrhage. In 2020, it was shown in zebrafish that pharmacologic MEK inhibition can not only prevent the formation of KRAS induced brain AVMs, but also prompt their regression (76). In 2021, it was shown in mice that pharmacologic MEK inhibition can prevent the formation of KRAS induced brain AVMs (75, 80). Just this year, in 2024, it was shown in mice that either pharmacologic KRAS or MEK inhibition may potentially cause brain AVM regression (74, 81).

While much work remains to further assess whether KRAS/MEK pathway inhibition can truly offer hemorrhage risk reduction or induce human brain AVM regression, there is reason for optimism when we look at early results of MEK inhibition in the context of extracranial sporadic AVMs. Similar to intracranial AVMs, in 2017 it was found that the majority of extracranial AVMs carry somatic KRAS/MEK pathway mutations (82, 83). KRAS and MEK inhibition have subsequently been found to induce regression of extracranial AVMs in humans and there are currently two phase II clinical trials in the United States investigating MEK inhibition for extracranial AVMs (81, 84). Additionally, a group in Canada is investigating the effect of 60 days of MEK inhibition on intracranial AVM architecture for patients already scheduled for microsurgical resection of their lesions (NCT06098872).

The development of novel targeted therapeutics of the KRAS/MEK pathway is an active area of investigation (77, 85, 86). Tremendous progress has been made over the past decade since trametinib, the first MEK inhibitor, received Food and Drug Administration (FDA) approval in 2013 for the treatment of *v-Raf murine sarcoma viral oncogene homolog B* (*BRAF*) mutated metastatic melanoma (87). In recent years direct KRAS inhibitors have been developed. Sotorasib was the first KRAS inhibitor to receive FDA approval for the treatment of *KRAS* G12C mutated non-small cell lung cancer (88). Additional mutant allele specific *KRAS* inhibitors, such as MRTX1133 for the treatment of *KRAS* G12D mutated solid tumors, are in clinical trials (89, 90). The application of KRAS/MEK pathway inhibition for the treatment of sporadic brain AVMs can benefit from lessons learnt from their use in the context of cancer. Namely, resistance can develop to single agent KRAS/MEK pathway inhibition and combination therapies may offer more durable efficacy (85, 86, 89).

## Discussion

The majority of sporadic brain AVMs are driven by somatic activating *KRAS* mutations in brain vascular endothelial cells. Targeting the KRAS/MEK pathway represents a promising therapeutic option for patients with sporadic brain AVMs.
